# Proteogenomics of the novel *Dehalobacterium formicoaceticum* strain EZ94 highlights a key role of methyltransferases during anaerobic dichloromethane degradation

**DOI:** 10.1007/s11356-023-28144-1

**Published:** 2023-06-10

**Authors:** Kenneth Wasmund, Alba Trueba-Santiso, Teresa Vicent, Lorenz Adrian, Stéphane Vuilleumier, Ernest Marco-Urrea

**Affiliations:** 1 Division of Microbial Ecology, Centre for Microbiology and Environmental Systems Science, University of Vienna, Vienna, Austria; 2 School of Biological Sciences, University of Portsmouth, Portsmouth, UK; 3 Departament d’Enginyeria Química, Biològica i Ambiental, Universitat Autònoma de Barcelona (UAB), Carrer de les Sitges s/n, 08193 Cerdanyola del Valles, Spain; 4 Current address: Department of Chemical Engineering, CRETUS Institute, Universidade de Santiago de Compostela, 15782 Santiago de Compostela, Galicia Spain; 5 Department Environmental Biotechnology, Helmholtz Centre for Environmental Research–UFZ, Leipzig, Germany; 6 Chair for Geobiotechnology, Technische Universität Berlin, Berlin, Germany; 7 Université de Strasbourg, CNRS, GMGM UMR 7156, Génétique Moléculaire, Génomique, Microbiologie, Strasbourg, France

**Keywords:** *Dehalobacterium*, Anaerobic dichloromethane degradation, Methyltransferases, Wood-Ljungdahl pathway, Shotgun proteomics

## Abstract

Dichloromethane (DCM, methylene chloride) is a toxic, high-volume industrial pollutant of long-standing. Anaerobic biodegradation is crucial for its removal from contaminated environments, yet prevailing mechanisms remain unresolved, especially concerning dehalogenation. In this study, we obtained an assembled genome of a novel DCM-degrading strain, *Dehalobacterium formicoaceticum* strain EZ94, from a stable DCM-degrading consortium, and we analyzed its proteome during degradation of DCM. A gene cluster recently predicted to play a major role in anaerobic DCM catabolism (the *mec* cassette) was found. Methyltransferases and other proteins encoded by the *mec* cassette were among the most abundant proteins produced, suggesting their involvement in DCM catabolism. Reductive dehalogenases were not detected. Genes and corresponding proteins for a complete Wood-Ljungdahl pathway, which could enable further metabolism of DCM carbon, were also found. Unlike for the anaerobic DCM degrader “*Ca*. F. warabiya,” no genes for metabolism of the quaternary amines choline and glycine betaine were identified. This work provides independent and supporting evidence that *mec*-associated methyltransferases are key to anaerobic DCM metabolism.

**Supplementary Information:**

The online version contains supplementary material available at 10.1007/s11356-023-28144-1.

## Introduction

Dichloromethane (DCM, methylene chloride) is produced by human activities (predominantly for industrial use as a solvent) and also naturally (e.g., volcanoes, oceanic emissions, and biomass burning) (Kolusu et al. 2018). It is classified as toxic and probable carcinogenic to humans (Schlosser et al. 2015), and it is also a potent ozone-depleting chemical which is not covered by the Montreal Protocol (Hossaini et al. 2017). DCM ranks eleventh on the European Union priority list of pollutants, and 91^st^ on the 2022 ATSDR Priority List of Hazardous Substances (ATSDR 2022). It is frequently detected as a groundwater pollutant following accidental or intentional spills and can migrate through the unsaturated soil column to accumulate at the bottom of the aquifer as dense non-aqueous phase liquid. The feasibility of removing DCM under oxic conditions has been widely investigated for facultative methylotrophs, which transform DCM via a glutathione *S*-transferase dehalogenase (Muller et al. 2011). Anaerobic microbial degradation of DCM has also been reported under nitrate-reducing and methanogenic conditions (Freedman and Gossett 1991; Freedman et al. 1997).

A strictly anaerobic bacterial strain capable of fermentation of DCM, *Dehalobacterium formicoaceticum* strain DMC, was reported in the 1990s (Mägli et al. 1995, 1996, 1998), but its genomic characterization was addressed only recently. To date, three anaerobic DCM-degrading bacteria, all belonging to the *Peptococcaceae* family, have now been genome-sequenced: *Dehalobacterium formicoaceticum* strain DMC (Chen et al. 2017); “*Candidatus* Dichloromethanomonas elyunquensis” from mixed culture RM (Chen et al. 2018; Kleindienst et al. 2019); and “*Candidatus* Formimonas warabiya” (formerly referred to as strain DCMF) (Holland et al. 2019, 2021)*.* DCM is the only known carbon and energy source for *Dehalobacterium formicoaceticum*. In contrast, strain “*Ca.* F. warabiya” is also capable of growing with non-chlorinated substrates (i.e., methanol, choline, and N,N,N-trimethylglycine) (Holland et al. 2021). Similarly, the mixed culture RM containing “*Ca.* D. elyunquensis” can also grow with dichloroacetate (Chen et al. 2021). The prevailing hypothesis is that all three known anaerobic DCM-degrading bacteria metabolize DCM via the Wood-Ljungdahl pathway encoded by their genomes (Holland et al. 2019; Chen et al. 2017; Kleindienst et al. 2019). However, DCM metabolism may proceed through two distinct routes of the Wood-Ljungdahl pathway, (i) one allowing mineralization of DCM to CO_2_ and H_2_, as in “*Ca*. D. elyunquensis”, and (ii) the other involving DCM fermentation, to acetate and formate in *D. formicoaceticum* and to acetate only in “*Ca.* F. warabiya” (Holland et al. 2021; Chen et al. 2020).

Another major, distinctive, and elusive feature of the three aforementioned bacteria is the enzyme responsible for transformation of DCM. For *D. formicoaceticum*, early physiological and biochemical studies with cell-free extracts as a catalyst showed that DCM reacts with tetrahydrofolate (H4F) to yield 5,10-methylene-H4F, which is then channeled into the Wood-Ljungdahl pathway (Mägli et al. 1996). The presence of numerous predicted methyltransferase genes in the genomes of *D. formicoaceticum* and “*Ca.* F. warabiya,” together with the lack of reductive dehalogenases in these strains, led to the hypothesis that methyltransferases could play a key role in DCM metabolism (Holland et al. 2019; Chen et al. 2017). In proteogenomic studies of “*Ca*. D. elyunquensis,” many methyltransferases were also found, as well as three reductive dehalogenases, two of which were detected in proteomic analyses of cultures using DCM as growth substrate (Kleindienst et al. 2019). Distinct enzymatic mechanisms for DCM dechlorination in the three different DCM-degrading bacterial systems were suggested by the different dual C-Cl isotope fractionation patterns observed for “*Ca*. D. elyunquensis” and *Dehalobacterium-*containing cultures (Blazquez-Palli et al. 2019; Chen et al. 2018).

Recently, a gene cluster annotated as *mec* (methylene chloride catabolism) and encoding various conserved methyltransferases and associated proteins was reported specifically among genomes of known anaerobic DCM-degrading bacteria, and not among other bacteria (Murdoch et al. 2022). Several methyltransferases and associated proteins of the *mec* cluster were among the most highly produced proteins in *D. formicoaceticum* and “*Ca*. D. elyunquensis” cultures grown with DCM, and copy numbers of *mecE* and *mecF* genes in groundwater correlated with DCM contamination levels (Murdoch et al. 2022). Together, these data suggested a key role of the proteins encoded by the *mec* cluster in DCM degradation.

To date, *D. formicoaceticum* is the only reported DCM-fermenting isolate (Mägli et al. 1996), since “*Ca*. D. elyunquensis” and “*Ca.* F. warabiya” are mixed cultures (Holland et al. 2019, 2021; Kleindienst et al. 2019). A novel DCM-degrading *Dehalobacterium*-containing enrichment culture from a membrane bioreactor operating at an industrial wastewater treatment plant was recently obtained and characterized (Trueba-Santiso et al. 2017). Efforts to isolate the *Dehalobacterium* strain from this culture were unsuccessful, and 16S rRNA gene amplicon sequencing confirmed the presence of *Dehalobacterium* together with three additional phylotypes belonging to *Acetobacterium*, *Desulfovibrio*, and *Wolinella* (Trueba-Santiso et al. 2017). Failure to obtain a DCM-degrading isolate was attributed to strict growth requirements of the *Dehalobacterium* strain, notably the need for a hydrogen scavenging partner, and nutrient supply from other bacteria. This dependency also indicates the importance of strong synergistic interactions in natural attenuation of DCM at contaminated sites. Detailed physiological studies of the *Dehalobacterium*-containing culture showed that DCM was transformed by three metabolic modules: (i) DCM fermentation, (ii) formate transformation to CO_2_ and H_2_, and (iii) H_2_/CO_2_-fueled acetogenesis. The *Dehalobacterium* strain in the culture studied in the present work was shown to only participate in DCM fermentation to acetate and formate (Trueba-Santiso et al. 2020).

In this study, we demonstrated the stability of this *Dehalobacterium*-containing DCM-degrading mixed culture and obtained a draft genome assembly of its *Dehalobacterium* strain by a hybrid Illumina/PacBio sequencing strategy, representing the second genome of this genus and species to date. We used this genome information to enable shotgun proteomic analysis of the DCM-degrading mixed culture and obtained new evidence for the *Dehalobacterium* proteins involved in growth with DCM.

## Materials and methods

### Cultivation of the *Dehalobacterium*-containing culture

The *Dehalobacterium*-containing mixed culture was maintained for more than two years by routine transfers in 100 mL glass serum bottles with 65 mL of anoxic synthetic medium modified from Trueba-Santiso et al. 2017. Briefly, this medium contained vitamins, trace elements, 22.8 μM tungsten, 24.2 μM selenium, and 200 mg/L of yeast extract (no acetate, pyruvate, fumarate, or formate). As a reducing agent, Na_2_S·9H_2_O and L-cysteine (0.2 mM each) were included. The medium was buffered with sodium bicarbonate (pH=7) and resazurin (10 mg/L) was added as a redox indicator. Medium was bottled inside an anoxic tent (Coy Laboratories, USA) in an atmosphere composed of N_2_ and 1–3% H_2_. Serum bottles were sealed with Teflon-coated butyl rubber septa and aluminum crimp caps. Bottles were autoclaved at 121°C for 40 min and then gassed with N_2_ to 0.4 bar overpressure. Neat DCM was added through the septa with a μL-glass syringe (Vici) to achieve the desired concentration in the serum bottles. Microcosms were statically incubated in the dark at 25°C in a thermostatized chamber and monitored periodically for chlorinated methanes via headspace gas sampling. Active cultures were transferred into fresh medium (3–7% v/v inoculum) during exponential DCM-degradation phase through the septum, maintaining anoxic sterile conditions. Cultures used in this study corresponded to the 50^th^ generation of this culture line and were sacrificed after consumption of 3.5 mM DCM.

### DNA extraction

Six parallel cultures grown under the same conditions as above were used for genomic analyses. Cells from each culture were pelleted by centrifugation at 7000 g and 10°C and resuspended in 1 mL of sterile PBS buffer. Bulk DNA was then extracted separately from each sample using the Gentra Puregene Yeast/Bacterial DNA extraction kit (Qiagen) following the manufacturer’s instructions. DNA samples were resuspended in 100 μL sterile Tris-HCl buffer (10 mM, pH 8) at 4°C under static conditions to minimize DNA fragmentation. DNA was quantified with a Qubit fluorimeter (ThermoFischer). DNA concentrations were in the range of 27.4–48.8 ng·μL^−1^.

### Microbial community profiling

The taxonomy of the microbial populations in the community of each sample was determined by sequencing the V4-V5 region of 16S rRNA genes. Briefly, PCR amplification of the 16S rRNA gene was carried out using 5 ng of genomic DNA as template using proprietary 192 barcoded primers (Metabiote® MiSeq Primers, GenoScreen, Lille, France) at final concentrations of 0.2 μM and an annealing temperature of 50°C for 30 cycles. PCR products were purified with Agencourt AMPure XP-PCR Purification system (Beckman Coulter, Brea, USA), quantified according to the manufacturer’s protocol, and mixed to equal concentrations. Sequencing was performed using a 250-bp paired-end sequencing protocol on the Illumina MiSeq platform (Illumina, San Diego, USA) at GenoScreen, Lille, France. Demultiplexing was done with CASAVA (Illumina). Numbers of reads ranged from 28,488 to 32,398 between samples. Raw paired-end reads were quality-filtered (Schmieder and Edwards 2011), read merging was done with FLASH (Magoč and Salzberg 2011), and forward and reverse primer sequences were removed using CutAdapt, with no mismatches allowed in primer sequences. After pretreatment, chimeras were removed with a proprietary protocol based on Usearch 6.1. Sequences were grouped at 97% identity into operational units (OTUs) with Uclust v1.2.22q (Edgar 2010). Taxonomic assignments of OTUs were obtained with the naive Bayesian classifier (v2.2) method (Wang et al. 2007) and the Greengenes database (version 13_8; www.greengenes.gov) (Wang et al. 2007).

### Sequencing, metagenome assembly, and annotations

Library preparations from DNA of the DCM-degrading consortium and sequencing using Illumina and PacBio technologies were performed by Genoscreen (Lille, France). The Illumina library was prepared with the Nextera XT sample prep kit (Illumina) and sequenced in a paired-end 2x100 bp HiSeq 2500 rapid run. Illumina sequence reads for genome assembly were trimmed of adapters using AdapterRemoval and default settings (Schubert et al. 2016). Reads were further trimmed for quality using a custom python3 script (https://github.com/kwasmund/Trim-Illumina) and the command line Illumina_trim.py-f Dhb2.pair1.truncated.fastq-r Dhb2.pair2.truncated.fastq-o reads.filtered-q 15-m 50-b 8. For PacBio, DNA fragmentation and ligation of single-molecule real-time (SMRT) adaptors was followed by BluePippin size selection (Sage Science) set at 4 kb, and PacBio sequencing was performed on one SMRT cell using PacBio RSII technology with P6 chemistry. Quality control was done using FastQC v0.11.5 (Andrews 2010).

An initial hybrid assembly was obtained using Unicycler (v 0.4.8) (Wick et al. 2017) with default settings using the quality-controlled Illumina paired-end sequences and PacBio sequences. The resulting contigs were binned using MetaBAT 2 (v 2.15) (Kang et al. 2019) with default settings for automatic binning. The *Dehalobacterium* genome bin was identified by BLASTX of contigs against the NCBI-nr database. Quality-controlled PacBio sequences from the original unbinned data were then mapped to the draft *Dehalobacterium* bin using minimap2 (v 2.17) (Li 2021) with default settings, and mapped sequences were collected and converted to fastq files using SAMtools (v 1.12) (Li et al. 2009). Collected PacBio sequences were then reassembled with all quality-controlled Illumina sequences using Unicycler and automatically binned with MetaBAT 2 as described above. Additional mapping of Illumina sequences to the initial draft *Dehalobacterium* bin, or by further iterations of PacBIO sequence mapping to the draft *Dehalobacterium* bins and/or reassembly, did not result in further assembly improvement. The resulting *Dehalobacterium* genome bin was automatically annotated using the RAST server (Aziz et al. 2008) in “classic” mode. Manual BLASTP analysis of selected protein sequences derived from the 19 obtained contigs against the NCBI-nr database gave best hits to *D. formicoaceticum* strain DMC for all contigs, suggesting the absence of contaminating contigs. Genome bin quality was assessed using CheckM (v1.1.1) (Parks et al. 2015). Additional automatic gene and protein annotations were performed on the MAGE MicroScope platform (Vallenet et al. 2020). Average Nucleotide Identity (ANI) analyses were conducted with the JSpeciesWS server in BLASTN mode (“ANIb”) (Richter et al. 2016). “Methyltransferase” proteins were subject to eggNOG-mapper (v2.0.0) (Cantalapiedra et al. 2021) using default settings (minimum hit e-value 0.001, minimum hit bit-score 60, minimum % of identity 40, minimum % of query coverage 20) with “-m diamond” option. They were also analyzed for conserved domains using the Conserved Domain search tool (Marchler-Bauer and Bryant 2004) against the Conserved Domain Database (CDD) (Lu et al. 2020) using default settings and a default e-value of 0.01. A phylogenomic tree was made on the IQ-TREE web-server using automatic substitution model selection (best-fit model LG+F+I+G4) and ultrafast bootstrapping (1000×) (Nguyen et al. 2015). The underlying alignment consisted of concatenated protein sequences derived from single copy marker genes retrieved from CheckM.

### Protein extraction

Triplicate samples consisting of two pooled microcosms each were used for proteome profiling. Cultures were centrifuged at 7000 g at 10°C for 20 min. The supernatant was discarded, and pelleted cells were washed with PBS and resuspended in 1 mL PBS. Cells were lysed by the freeze-thaw method (−20°C/+20°C, 6x) and then sonicated for 30 s. Samples were then concentrated (10 kDa cut-off Amicon Ultra–0.5 mL Centrifugal Filters, Millipore) by centrifugation at 14000 g for 30 min at 10°C. Filters were inverted, and the protein extract was spun-off for 2 min at 1000 g, 10°C. Protein concentrations in these samples were estimated using the Bradford method. The three samples were then further processed in parallel.

### Nano-liquid chromatography tandem mass spectrometry (nLC-MS/MS)

Protein samples were treated as described previously to reduce disulfide bridges and alkylate cysteine residues (Seidel et al. 2018). Protein digestion was performed by addition of 0.1 μg of porcine trypsin (Proteomics Sequencing Grade, Promega) and overnight incubation at 37°C on a shaker. The obtained peptides were dried, resolubilized in 0.1% formic acid, and desalted using Zip Tip-μC18 material (Merck Millipore). Peptides were analyzed by nano-liquid chromatography tandem mass spectrometry (nLC-MS/MS) on a nanoUPLC system (nanoAcquity, Waters) hyphenated via a TriVersa NanoMate (Advion, Ltd., Harlow, UK) to an Orbitrap Fusion mass spectrometer (Thermo Scientific) as described previously (Seidel et al. 2018). Glyceraldehyde-3-phosphate dehydrogenase (GapDH) from *Staphylococcus aureus* was added as internal standard before sample processing. Peptide identification was conducted by Proteome Discoverer (v2.2, Thermo Fisher Scientific) using SequestHT as a search engine and a fasta file of proteins from the draft genome of the *Dehalobacterium* strain EZ94 as the database. A false discovery rate threshold of 1%, defined versus a decoy database (Target Decoy PSM evaluator implemented in Proteome Discoverer) was set for peptide identification. Label-free quantification of detected proteins was performed with Minora node implemented in Proteome Discoverer on the basis of area counts of corresponding peptide peaks in precursor scans.

## Results

### Sequencing of a stable DCM-degrading consortium and analysis of the dominant *Dehalobacterium* strain

In a previous study, we showed that our DCM-degrading anaerobic mixed culture was composed mainly of bacteria belonging to the genera *Dehalobacterium*, *Desulfovibrio*, *Acetobacterium*, and *Wolinella* (Trueba-Santiso et al. 2020). The 16S rRNA gene amplicon data confirmed that the DCM-degrading consortium was dominated by a *Dehalobacterium* population (87.1%±1.9 relative abundance), with lower relative abundances of the genera *Acetobacterium* (7.6%±1.2), *Desulfovibrio* (4.7%±1.0), and *Wolinella* (0.6%±0.1) (Table S1). The very low variability observed between biological replicates shows the stability of the consortium under the selective conditions used.

**Fig. 1:**
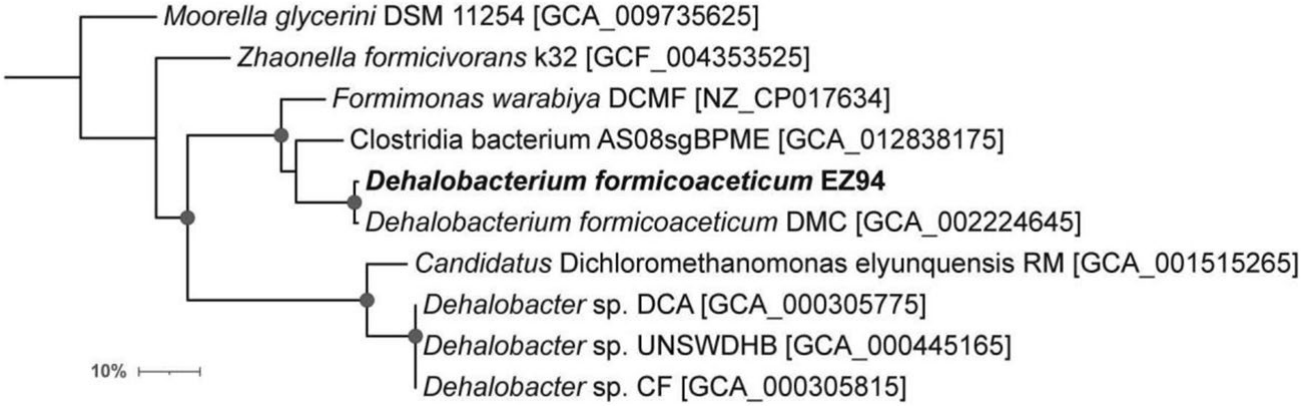
Phylogenomic tree based on proteins from 34 single copy marker gene genes derived from CheckM analysis (see the “Materials and methods” section). Circles on nodes indicate bootstrap values >90%. The scale bar represents 10% sequence divergence

Metagenomic sequencing on DNA of the consortium culture performed using Illumina and PacBio techniques, followed by genome binning of the obtained contigs, allowed recovery of a draft genome of the dominant *Dehalobacterium* population. It consisted of 3,650,560 bp in 19 contigs with a G+C content of 43.2%. This is closely similar to the reported genome of *D. formicoaceticum* strain DMC of 3,766,545 bp and has identical G+C content (Chen et al. 2017, NCBI accession number GCA_002224645.1). The draft genome of our *Dehalobacterium* strain displayed an ANIb value of 98.6% to the *D. formicoaceticum* strain DMC genome, with 93% alignment. The genome was estimated to be 95.7% complete from CheckM analysis, with 2% contamination, as reported for the complete circular genome of *D. formicoaceticum* strain DMC. This suggests that CheckM missed and/or misassigned marker genes from these organisms and that the genome of the *Dehalobacterium* strain from our DCM-degrading consortium is near-complete. Close similarity of our strain to strain DMC was also supported by phylogenomic analysis of 34 single copy marker proteins (Fig. 1). Further, and as for strain DMC, six 16S rRNA gene copies assigned to *Dehalobacterium* were identified in an initial draft genome, which, however, were not recovered in our improved final hybrid genome assembly. On the basis of this high similarity of our strain to *D. formicoaceticum* DMC and of previous recommendations that strains with ANI similarity >95% should be assigned to the same species (Goris et al. 2007), we defined the strain in our DCM-degrading consortium as *D. formicoaceticum* strain EZ94, a new strain of the species *D. formicoaceticum*.

### Genomic analysis of *Dehalobacterium* formicoaceticum strain EZ94

**Fig. 2:**
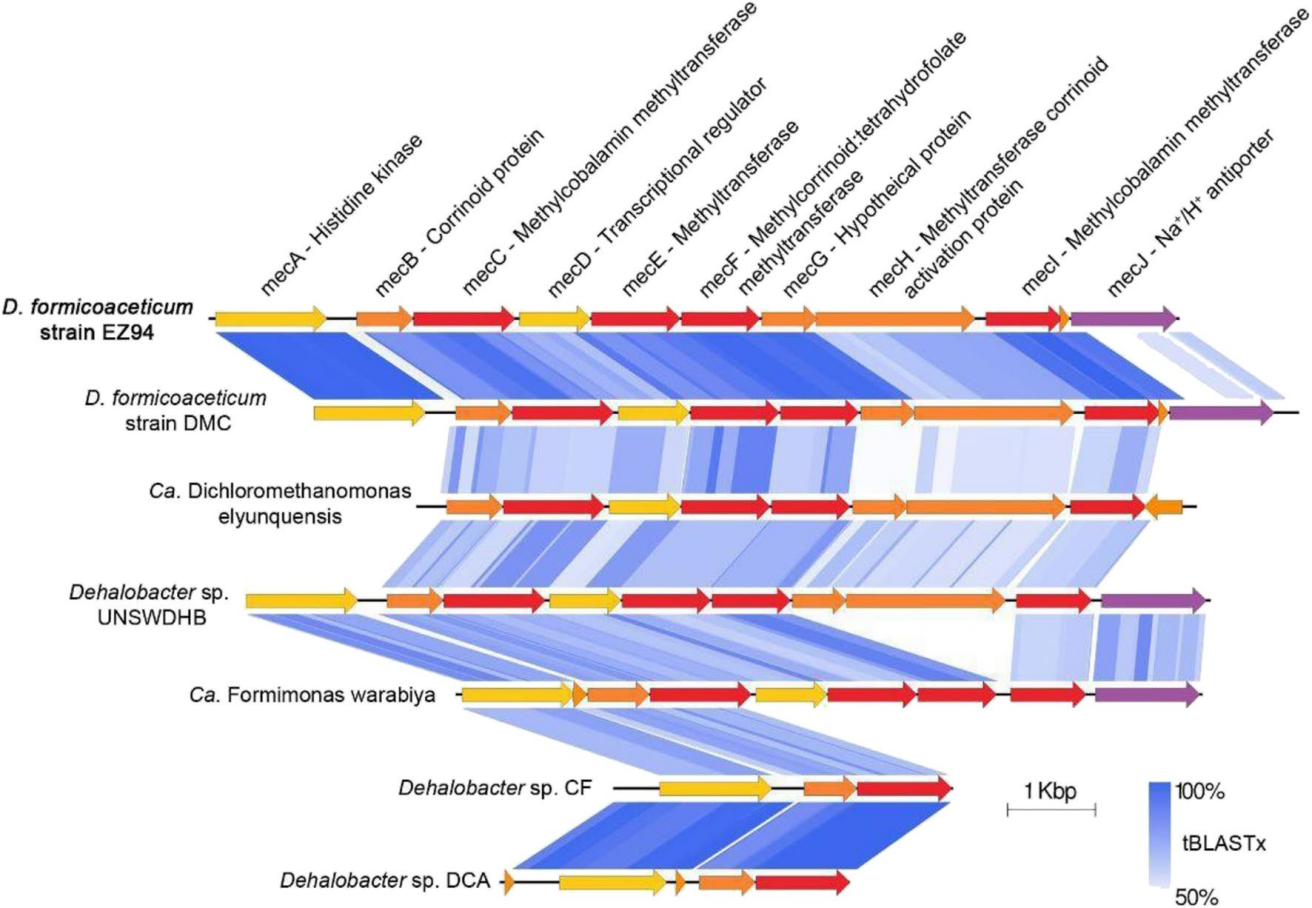
Gene synteny for the *mec* gene cluster among DCM-degrading bacteria. Blue shading represents tBLASTx identity values between corresponding proteins

The genome of strain EZ94 harbors a gene cluster corresponding to the conserved methylene chloride catabolism (*mec*) gene cassette encoding a multiple methyltransferase system recently shown to be associated with anaerobic DCM degradation (Murdoch et al. 2022). This gene cluster (DHBM_v1_160003 to DHBM_v1_160013; in the following, the locus tag “DHBM_v1_” is omitted for clarity) encodes 10 proteins MecA to MecJ with high sequence amino acid identity (average 91.3%) to corresponding proteins from other DCM degraders (Fig. 2 and Table 1). An open reading frame for one additional small (37 amino acid) hypothetical protein (160012) between genes for MecI and MecJ was also defined, unlike in other anaerobic DCM-degrading bacteria except for *D. formicoaceticum* strain DMC (Table 1). Notably, no reductive dehalogenase genes were detected in the genome.

**Table 1:**
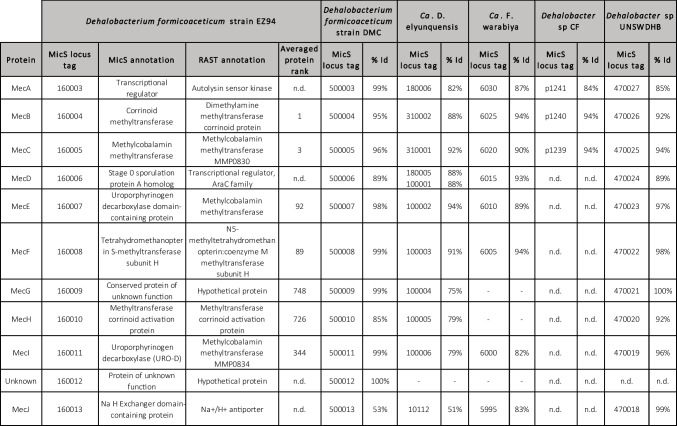
Percentage identity of proteins encoded by the *mec* gene cassette of strain EZ94 to their homologs in other anaerobic DCM-degrading organisms. *Dehalobacter* sp. CF and *Dehalobacter* sp. UNSWDHB were also included for reference. Averaged protein abundance and ranking are derived from Table S4. “n.d.,” not detected. The MicroScope (MicS) gene locus tag identifiers were provided omitting “DHBM_v1_,” “DEFO_v1_,” “LINDB_v1_,” “DCMF_,” “DCF50_,” and “AUUR_v1_” in *Dehalobacterium* spp. strains EZ94, DCM, “*Ca*. D. elyuquensis,” and “*Ca*. F. warabiya,” and *Dehalobacter* strains CF and UNSWDHB for clarity, respectively

In this context, we also searched the automatically annotated genome for proteins with “methyltransferase” annotations and classified them further according to conserved domains from CDD searches and by mapping to the eggNOG database (eggNOG-mapper). Apart from Mec proteins, these identified 124 genes associated with C1 transfer, including 25 diversely annotated as mono-, di-, or trimethylamine:corrinoid methyltransferases (Table S2).

**Fig. 3:**
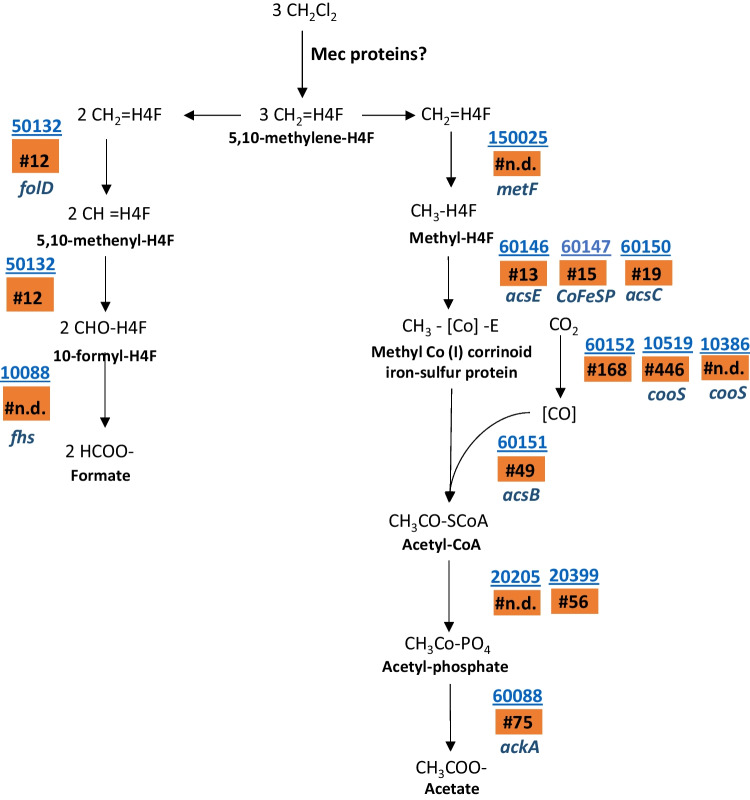
Proposed pathway for metabolism of DCM by *D. formicoaceticum* strain EZ94. Gene locus identifier numbers for corresponding enzymes are underlined, with mean protein abundance rank (“#” in orange boxes; “n.d.,” not detected, see Table S4). *folD*, methylene-H4F dehydrogenase/cyclohydrolase; *fhs*, formate-H4F ligase; *metF*, methylene-H4F reductase; *acsE*, methyltransferase; *CoFeSP*, methyl-Co(I) corrinoid iron sulfur protein; *cooS*, CO dehydrogenase; *acsB*, acetyl-CoA synthase; *pta*, phosphate acetyltransferase; *ackA*, acetate kinase

The genome of strain EZ94 also encodes a complete set of genes for the Wood-Ljungdahl pathway (Fig. 3). This is in accordance with previous reports of anaerobic DCM-degrading bacteria and indicates strain EZ94 has the capacity to metabolize DCM through this pathway. In particular, all proteins in the Wood-Ljungdahl pathway were almost identical (92.83 to 100% amino acid identity) with those in *D. formicoaceticum* strain DMC. In this context, biosynthesis pathways for essential cofactors folate, heme, riboflavin, cobalamin, pantothenate, and coenzyme A are fully or almost fully encoded in the genome of strain EZ94 (Table S3). The biosynthesis pathway for biotin appears to be absent in all anaerobic DCM degraders. As an intriguing difference among anaerobic DCM degraders, the menaquinone biosynthesis pathway is absent in strain EZ94, as in *D. formicoaceticum* strain DMC and *Ca*. F. warabiya, but almost fully encoded in “*Ca*. D. elyunquensis” (Table S3). However, all DCM-degrading strains encode two genes that may catalyze the last steps of menaquinone biosynthesis (1,4-dihydroxy-2-naphthoate octaprenyltransferase (10507) and demethylmenaquinone methyltransferase (10199), Table S3). Additionally, genes for twelve subunits of the proton-pumping NADH:ubiquinone oxidoreductase complex were detected in the genome of strain EZ94 (NuoABCDFHIJMNLK).

### Proteome profile of strain EZ94 in the DCM-degrading consortium

Shotgun proteomic analyses were performed for three replicate cultures of the DCM-degrading consortium grown on DCM for 50 consecutive transfers and resulted in the identification of 855 distinct proteins of *D. formicoaceticum* strain EZ94 (Table S4), accounting for 23% of proteins defined from the genome sequence.

All proteins of the *mec* cassette were detected with the exception of MecA, MecD and MecJ (Table 1). These three proteins are predicted to be regulatory proteins and/or to be membrane-associated. This may help explain why they were not detected experimentally. The small predicted ORF between MecI and MecJ was not detected either. Proteomic analysis further revealed that MecB, annotated as corrinoid methyltransferase (160004), was the most abundant protein in the three cultures analyzed (Table S4). The third most abundant protein was MecC, annotated as methylcobalamin methyltransferase (160005) (Table S4). These two protein products of the *mec* gene cluster display protein identities of 88–95% and 92–96% with MecB and MecC from DCM-degrading *D. formicoaceticum* DMC, “*Ca.* D. elyunquensis” and “*Ca.* F. warabiya”, respectively (Table 1). Worthy of note, other related proteins in databases show only low levels of identity (<54.9% for MecB and <40.4% for MecC), suggesting these methyltransferases are specific to DCM-degrading bacteria of the Peptococcaceae.

The second most abundantly produced protein (50017) is a 131 amino acid long protein unlike any well-characterized protein in databases, automatically annotated as pyridoxamine-5′-phosphate oxidase family protein with a potential flavin-mononucleotide binding domain. It was also the second most abundant protein found in the proteogenomic analysis of strain DMC (Murdoch et al. 2022). Nevertheless, it is not found in the other two DCM degraders “*Ca.* D. elyunquensis” or “*Ca.* F. warabiya,” suggesting that it may not be involved or essential for DCM degradation.

*D. formicoaceticum* strain DMC is acetogenic and suggested to metabolize the methyl group of DCM via the Wood-Ljungdahl pathway (Mägli et al. 1996). The Wood-Ljungdahl pathway can be used in either reductive (i.e., CO_2_ fixation) or oxidative directions (i.e., acetate oxidation). In strain EZ94, most of the proteins associated with the carbonyl and methyl branch of the Wood-Ljungdahl pathway were produced during growth with DCM (Fig. 3). Worthy of note, however, the protein product of a formate dehydrogenase subunit alpha (*fdhA*) gene (10437) was not detected.

Several Mec proteins, as well as enzymes of the Wood-Ljungdahl pathway, are associated with corrinoids or tetrahydrofolate. Among the full set of genes encoding for corrinoid and tetrahydrofolate biosynthesis in strain EZ94, 8 proteins associated with the upper corrinoid biosynthesis pathway starting from L-glutamate to ado-cobinamide and 3 proteins associated with nucleotide loop assembly of corrinoids were identified in the genome (Table S3). A predicted cobalt transporter (10141, CbiQ) and a corrinoid salvaging enzyme (20161, CbiZ) were also identified (Table S4). For tetrahydrofolate, six of the seven proteins involved in the transformation of guanosine 5′-triphosphate into 5,6,7,8-tetrahydrofolate were produced in at least one replicate, indicating biosynthesis of this cofactor.

With regard to heme biosynthesis, four proteins were detected in at least one replicate for the reactions from L-glutamyl-tRNA to coproporphyrinogen III. The genes for conversion of coproporphyrinogen III to heme via the classical pathway seemed to be encoded, but proteins were not detected. Similarly, and although several genes for the cytochrome *c* assembly pathway were detected in the genome, none of the corresponding proteins (subunits CcmA, CcmB, CcmC, CcmE, and CcmF) were detected by LC-MS/MS. Conversely and despite the fact that the biosynthesis pathway for menaquinone is lacking in strain EZ94, three subunits of the proton-pumping NADH:ubiquinone oxidoreductase complex (NuoC, NuoD, and NuoI) were detected in two replicate cultures. However, genome-encoded polyprenyltransferase (MenA, 10507) and methyltransferase (MenG, 10199) were not detected in proteomic analyses.

## Discussion

Metagenomic analysis of our DCM-degrading consortium confirmed that the dominant strain EZ94 belongs to the species *D. formicoaceticum*, that it also encodes the *mec* gene cassette recently reported to be associated with metabolism of DCM by *D. formicoaceticum* DMC, and that the Mec proteins are highly produced during growth with DCM (Table S4). Comparative genomics suggest that no close homologs of the *mec* gene cassette are found in publicly available bacterial genomes aside from *D. formicoaceticum* strains, “*Ca.* D. elyunquensis” and “*Ca*. F. warabiya”, and among *Dehalobacter* strains that produce DCM during organohalide respiration with chloroform (Table 1, Deshpande et al. 2013, Tang et al. 2016). High amounts of Mec proteins were also detected in *D. formicoaceticum* and “*Ca.* D. elyunquensis” growing with DCM (Murdoch et al. 2022). Specifically, corrinoid-associated methyltransferases MecB, MecC, MecE, and MecF were the most abundant proteins among the *mec* gene cluster in all three organisms, with MecB the most highly produced. Early experiments with cell extracts of *D. formicoaceticum* DMC had already shown a requirement for methyl viologen for dehalogenation activity and light-reversible propyl iodide inhibition of DCM degradation, suggesting the involvement of corrinoids (Mägli et al. 1996). Worthy of note, the genome of strain EZ94 also harbors a full set of genes for a complete corrinoid biosynthetic pathway, with several of the corresponding proteins detected by mass spectrometry (Table S4).

Nevertheless and despite the high sequence similarity found for Mec proteins between *Dehalobacterium* strains, “*Ca.* F. warabiya” and “*Ca.* D. elyunquensis” (Table 1), dual C and Cl isotope fractionation analysis previously indicated that different anaerobic DCM-degrading strains from different genera catalyze mechanistically distinct C-Cl bond cleavage reactions (Chen et al. 2018; Blazquez-Palli et al. 2019). Interestingly and in contrast to the two *Dehalobacterium formicoaceticum* strains, “*Ca.* D. elyunquensis” also encodes reductive dehalogenases, two of which were produced during growth with DCM (Kleindienst et al. 2019). This suggests that “*Ca.* D. elyunquensis” may be unique among known anaerobic DCM-degraders in using reductive dehalogenases for DCM dehalogenation, but this has not yet been demonstrated experimentally. Rather, Murdoch et al. (2022) proposed that for strain DMC as well as for “*Ca.* D. elyunquensis,” C1 carbon metabolism from DCM first involves methyltransferase MecE that would eliminate one chlorine substituent from DCM with subsequent chloromethyl transfer to MecB, yielding a chloromethyl corrinoid protein that would be further transformed to methylene-H4F by MecF. However, this leaves the potential role of other corrinoid-associated proteins MecC and MecI encoded by the *mec* cassette that are also produced during growth with DCM unexplained. In particular, MecE is produced at similar levels as MecB and about 25x higher than MecC. Protein separation and/or heterologous expression coupled with activity assessment will be needed to identify which of the specific Mec proteins are involved in the different steps of DCM dechlorination.

Pathways for biosynthesis of cobalamin, menaquinone, and heme differ among the anaerobic DCM-degrading strains. Specifically, cobalamin biosynthesis appears incomplete in “*Ca*. D. elyunquensis,” whereas almost all genes for both upper and lower pathways for *de novo* biosynthesis of cobalamin are found in *D. formicoaceticum* and “*Ca*. F. warabiya” strains, with 11 of the corresponding proteins of strain EZ94 detected by mass spectrometry in at least one replicate culture (Table S3). Conversely, the biosynthesis pathway for the electron carrier menaquinone is found in “*Ca*. D. elyunquensis,” with two proteins detected during growth with DCM (Kleindienst et al. 2019), but it was not detected in the other anaerobic DCM-degrading strains (Table S3). Also, heme biosynthesis in *Dehalobacterium* and “*Ca*. F. warabiya” strains likely proceeds via the classical pathway, whereas “*Ca*. D. elyunquensis” appears to use the alternative route to heme as described for Archaea and sulfate-reducing bacteria, in which heme is synthesized from uroporphyrinogen III via siroheme (Kühner et al. 2014).

With respect to carbon processing downstream of DCM dehalogenation, the genome of strain EZ94 encodes a complete Wood-Ljungdahl pathway, with most corresponding proteins experimentally detected, indicating that metabolism of DCM proceeds via this pathway after methylene-H4F formation (Fig. 3). Our genomic and proteomic data thus suggest that strain EZ94, as likely also strain DMC, is mixotrophic; i.e., it depends on both inorganic (CO_2_) and organic (DCM) carbon to grow. A requirement for CO_2_ in EZ94 is further supported by physiological studies showing that omission of CO_2_/HCO_3_ from the medium stopped DCM consumption (data not shown). Incidentally, this was also observed for “*Ca.* D. elyunquensis” (Kleindienst et al. 2019), indicating that although the dechlorinating enzymatic system of the two strains appears distinct, they both metabolize tetrahydrofolate-associated DCM carbon by the Wood-Ljungdahl pathway.

Although the genome encoded a formate dehydrogenase subunit alpha (10437), the protein was not detected in the proteome. Indeed, our *Dehalobacterium* strain died-out after several transfers when amended with formate instead of DCM as energy source (Trueba-Santiso et al. 2020). Interestingly, the almost identical (99.8% similarity) homolog of *D. formicoaceticum* strain DMC (accession WP_198306660) was detected in a previous proteomic study (abundance rank #287/2014, Murdoch et al. 2022), and “*Ca.* F. warabiya” also features a homolog of this gene (55% identity at the protein level). Nevertheless, *D. formicoaceticum* strain DMC and *Ca.* F. warabiya cannot grow with formate either. Therefore, the metabolic function of this formate dehydrogenase remains unknown.

*Dehalobacterium formicoaceticum* strains are indeed unique among anaerobic DCM-dechlorinating bacteria in that DCM fermentation is their only growth-supporting metabolism known so far. Other bacteria with the *mec* cassette associated with DCM utilisation appear to be metabolically more versatile. For instance, the mixed culture containing “*Ca*. D. elyunquensis” also ferments dichloroacetate to acetate, carbon dioxide, and hydrogen (Chen et al. 2021). In this case, dichloroacetate dehalogenation involves an (*S*)-2-haloacid dehalogenase not found in *D. formicoaceticum*. Interestingly, re-examination of published proteomic data (Chen et al. 2021) showed that MecB, MecC, MecE, MecF, MecH, and MecI proteins of “*Ca*. D. elyunquensis” were 56–71 times more abundant upon growth with DCM compared to growth with dichloroacetate, underlining the specific association of *mec* cassette proteins with DCM metabolism. Similarly, “*Ca*. F. warabiya” also grows with non-chlorinated substrates methanol, choline, and glycine betaine (Holland et al. 2021). Strain EZ94 contains several genes annotated as glycine/sarcosine/betaine reductase complexes (10155-10158, 20275-20277, 10725, and 10726), although no choline dehydrogenase or betaine aldehyde dehydrogenase associated with transformation of choline to betaine, or for demethylation of betaine or dimethylglycine, were identified. Similarly, a large number of genes diversely annotated as mono-, di-, or trimethylamine or methanol corrinoid methyltransferases (Table S2) were detected in the genome of strain EZ94. It is possible that alternative compounds or mixtures of compounds lacking C-C bonds such as those mentioned above may be found to support growth of strain EZ94 in the future.

## Conclusions

*Dehalobacterium formicoaceticum* was the dominant organism in our DCM-degrading consortium after growth for 2 years with DCM. The proteogenomic characterization of strain EZ94 reported here provides a second genome sequence for a member of the genus *Dehalobacterium* and for the species *Dehalobacterium formicoaceticum*, after that of strain DMC (Chen et al. 2017). Both strains grow exclusively by DCM fermentation as the sole energy source. As other anaerobic DCM degraders, the genome of strain EZ94 contains a *mec* gene cluster, confirming its potential as a DNA-level biomarker for evaluating intrinsic biodegradation potential of DCM at contaminated sites. Comprehensive proteome profiling of strain EZ94 in the consortium culture showed that proteins of the *mec* gene cluster were highly produced, in agreement with recent studies with *D. formicoaceticum* DMC and “*Ca.* D. elyunquensis.” Our findings thus independently confirm and extend growing insights into DCM fermentation and may contribute to decipher the still elusive mechanisms of anaerobic DCM degradation.

## Supplementary information

ESM 1 (XLSX 286 kb)

## Data Availability

Illumina and PacBio metagenome sequences, Illumina 16S rRNA gene sequences, and the draft genome are available under GenBank BioProject Accession PRJNA857723. The assembled genome is available at NCBI under Genbank accession number GCA_024705665. The final assembly, as well as a preliminary draft genome assembly also including 16S rRNA genes, is also available on the Microscope platform at https://mage.genoscope.cns.fr/microscope/mage/viewer.php?O_id=13191 and https://mage.genoscope.cns.fr/microscope/genomic/overview.php?O_id=11670, respectively.
